# Gene Regulatory Network Analysis of Decidual Stromal Cells and Natural Killer Cells

**DOI:** 10.1007/s43032-024-01653-1

**Published:** 2024-08-01

**Authors:** Kalle T. Rytkönen, Nigatu Adossa, Sebastián Zúñiga Norman, Tapio Lönnberg, Matti Poutanen, Laura L. Elo

**Affiliations:** 1 Turku Bioscience Centre, University of Turku, Åbo Akademi University, Turku, Finland; 2 Institute of Biomedicine, Research Centre for Integrative Physiology and Pharmacology, University of Turku, Turku, Finland; 3 InFLAMES Research Flagship Center, University of Turku, Turku, Finland; 4 Institute of Biomedicine, University of Turku, Turku, Finland

**Keywords:** Uterus, Endometrium, scRNA-seq, Gene regulatory network, Transcription factor

## Abstract

Human reproductive success relies on the proper differentiation of the uterine endometrium to facilitate implantation, formation of the placenta, and pregnancy. This process involves two critical types of decidual uterine cells: endometrial/decidual stromal cells (dS) and uterine/decidual natural killer (dNK) cells. To better understand the transcription factors governing the in vivo functions of these cells, we analyzed single-cell transcriptomics data from first-trimester terminations of pregnancy, and for the first time conducted gene regulatory network analysis of dS and dNK cell subpopulations. Our analysis revealed stromal cell populations that corresponded to previously described in vitro decidualized cells and senescent decidual cells. We discovered new decidualization driving transcription factors of stromal cells for early pregnancy, including DDIT3 and BRF2, which regulate oxidative stress protection. For dNK cells, we identified transcription factors involved in the immunotolerant (dNK1) subpopulation, including IRX3 and RELB, which repress the NFKB pathway. In contrast, for the less immunotolerant (dNK3) population we predicted TBX21 (T-bet) and IRF2-mediated upregulation of the interferon pathway. To determine the clinical relevance of our findings, we tested the overrepresentation of the predicted transcription factors target genes among cell type-specific regulated genes from pregnancy disorders, such as recurrent pregnancy loss and preeclampsia. We observed that the predicted decidualized stromal and dNK1-specific transcription factor target genes were enriched with the genes downregulated in pregnancy disorders, whereas the predicted dNK3-specific targets were enriched with genes upregulated in pregnancy disorders. Our findings emphasize the importance of stress tolerance pathways in stromal cell decidualization and immunotolerance promoting regulators in dNK differentiation.

**Supplementary Information:**

The online version contains supplementary material available at 10.1007/s43032-024-01653-1.

## Introduction

Successful human reproduction relies on the proper decidualization (differentiation) of the uterine endometrium. This process enables uterus to support embryo implantation, formation of the placenta, and the maintenance of pregnancy. Two key types of uterine cells play roles in this process: endometrial/decidual stromal cells (dS) and uterine/decidual natural killer (dNK) cells [1–5]. Previously, the transcription factors (TFs) and gene regulatory networks of these cell types have been studied through various in vitro and tissue imaging approaches. More recently, single-cell sequencing has provided unprecedented resolution for studying the subpopulations of these cell types directly from tissue extracted in vivo [1, 2], but this data has not been comprehensive explored with the latest gene regulatory network analysis methods.

For the decidualization of endometrial stromal cells, several essential transcription factors have been described in vitro, including PGR, HOXA10, HOXA11, FOXO1, and HAND2 [6–8]. In contrast, studies on core transcription factors regulating natural killer (NK) cell differentiation have mostly focused on their differentiation towards cytotoxic states [9], while less is known about the regulatory states of uterine or decidual NK cells. Recent research has shown that the differentiation process of stromal cells and NK cells is interconnected, and ligand-receptor interactions between these cell types have been studied from scRNA-seq data in both pregnant [1] and menstrual cycle states [2]. Notably, stromal cells drive dNK differentiation by secreting IL-15 [10] resulting in distinctive dNK populations, including an immunotolerance promoting dNK1 population [1]. On the other hand, dNK cells regulate stromal decidualization by actively clearing senescent decidual cells [3, 8] thus enhancing the differentiation process of fully decidualized cells.

Importantly, the proper differentiation of dS and dNK cell subpopulations is involved in the development of receptive uterine environment for implantation, including regulation of the growth of uterine spiral arteries, but also in the development and maintenance of pregnancy [11]. During 1st trimester of pregnancy the uterine dS and dNK populations continue to support spiral artery development and trophoblast invasion [11]. This is further evidenced by the observations that defects in the differentiation processes of these cell types are associated with disorders of pregnancy and the placentation, including recurrent pregnancy loss (RPL) [8, 12] and preeclampsia [13, 14].

Recent high quality single-cell data [1] has not yet been utilized for a robust computational analysis of gene regulatory networks (GRNs) of physiologically relevant in vivo cell states during pregnancy. One of the widely used methods for this purpose is the Single-Cell rEgulatory Network Inference and Clustering (SCENIC) method [15]. In our study, we for the first time employ the SCENIC approach to investigate decidual stromal and NK cell subpopulations during the first trimester pregnancy and identify transcriptional regulators specific to these subpopulations. This analysis helps to augment knowledge gained from previous cell type-specific in vitro studies and to predict both known and novel TFs that regulate the stromal and NK cells subpopulations in vivo. Furthermore, for translational insight on the discovered subpopulation specific TFs, we also studied how their predicted target genes are regulated in pregnancy-related disorders that are associated with decidual function during 1st trimester pregnancy, including RPL and preeclmapsia. By combining the predicted core TF target gene sets with transcriptomic data from these disorders, we predicted cell subpopulation-specific TF targets that are regulated in these conditions.

## Materials and Methods

### scRNA-seq Data and Clustering

The 1st trimester pregnancy selective termination scRNA-seq (10X Genomics) data consisted of 6 decidual samples stored with ArrayExpress accession E-MTAB-6701 [1]. The preprocessed scRNA-seq UMI counts were extracted for 12,584 stromal cells with dS1, dS2 and dS3 annotations and 11,884 natural killer cells with dNKp, dNK1, dNK2, and dNK3 annotations. We used standard workflow for scRNA-seq analysis with Scanpy [v1.8.2] [16], this included steps of normalization [sc.pp.normalize, sc.pp.log1p], scaling [sc.pp.scale, sc.tl.pca], batch correction over individual donors using Mutual Nearest Neighbours [mnnpy.mnn_correct], clustering [sc.pp.neighbors, sc.tl.leiden] and UMAP projections [sc.tl.umap,sc.pl.umap]. These steps were performed with default parameters with the exception of scanpy’s *Leiden* function with resolution of 0.3 for ‘dS’ and 0.2 for ‘dNK’ was used for clustering. The cluster markers were calculated from the Scanpy’s clustering output using Seurat’s FindMarkers function [v4.0] [17] with FDR < 0.05 and ranked by expression fold-change. This Scanpy pipeline was similar to the original authors used in Seurat [v3.2.2], which by default includes following functions: Normalization [NormalizeData], Clustering [FindClusters], Differential Expression Analysis [FindMarkers or FindAllMarkers], Data Integration (CCA) [RunCCA], Shared Nearest Neighbor (SNN) Graph Construction [FindNeighbors], lastly, UMAP projections [RunUMAP]. The clusters for stromal cells were annotated based on the original authors’ annotation and the newly identified cluster was annotated taking into account the previously in vitro defined senescent decidual cluster [18]. The cluster annotation for dNK cells was based on the original authors’ annotation together with the results of the gene regulatory network analysis. Overall the resulting main clusters from our Scanpy clustering and original authors’ Seurat [v3.2.2] clustering were concordant, so we continued to gene regulatory network analysis with our Scanpy clustering without further emphasis for comparison of the clusterings.

### Gene Regulatory Network Analysis

Gene regulatory network analysis was performed using pySCENIC [v0.11.2] [15]. We used raw count matrix as an input to calculate the adjacency matrix using *grnboost2* function. The *ctx* function was used for cis regulatory TF-motif enrichment analysis to identify direct targets using the human cisTarget motif database downloaded from https://resources.aertslab.org/cistarget/. The default setting with -500 + 100 bp TSS and ± 10 kb TSS databases was used for the analysis, so that both promoter and proximal enhancer binding sites were considered in the prediction of the target genes for the TFs. To determine the regulon specificity matrix, we used *aucell* function with *auc_threshold* value of 0.05 to score the activity of each regulon in each cell. Then, we used *binarize* function to specify regulon activity as a binary outcome (1 for on and 0 for off) in each cell. Finally, we used R package ComplexHeatmap (v2.6.2) for plotting the heatmap of binary regulon activity for each cell using the top 10 regulons ranked by the regulon specificity score (rss) for each subpopulation (RNA-seq clusters). Of these top regulons we further defined core cluster specific regulons by selecting the regulons that clustered together, and required that a given cluster contained at least one regulon with > 50 target genes. Additionally, for downstream analysis, we also selected IRX3 regulon, which had the highest rss value for dNK1 with 43 predicted targets, and IRF7, which was a shared dNK2/3 regulon based on rss and high mRNA expression in both dNK2 and dNK3 clusters. Cytoscape (v3.9.1) was used for the network visualization of specific regulons and their upregulated target genes in the particular scRNA-seq subpopulation (FDR < 0.05).

### Functional Enrichment Analysis with Gene Ontology

For each subpopulation, the predicted targets of the selected core regulons were combined for functional enrichment analysis. These combined regulon target gene lists were used as input for METASCAPE (https://metascape.org/) where Gene Ontology database was selected for analysis.

### Overrepresentation Analysis with Cell Type-Specific Pregnancy Disorder Datasets

To test the translational significance of our SCENIC analysis results, we intersected the predicted regulon target genes with cell type-specific differentially expressed genes from RPL and preeclampsia datasets. These conditions have previously been recognized to have changes in dS and dNK subpopulations and their states during early 1st trimester pregnancy [8, 11–14]. Statistical significance of the enrichment was determined using Fisher’s exact test in R, with all RefSeq protein-coding genes (n = 20,203) as the background. For natural killer cells, the cell type -specific datasets included two datasets of RPL: genes up- or down regulated in missed abortion (MA) by [19] and unexplained RPL associated cluster enriched/underrepresented genes [12] (list kindly provided by Prof. Lisha Mou). We also utilized early-onset (before week 34) (EOP) and late-onset (LOP) severe preeclampsia up- or down regulated genes [20] that were previously filtered to contain only the genes that also were cell type-specific markers to NK cells [14]. For stromal cells, we utilized the same preeclampsia data [20] and data from women with previous severe preeclampsia during late secretory menstrual cycle phase (PP-M) [13], both filtered by stromal markers [14].

## Results

### Reannotation of the Stromal Cluster Using scRNA-seq Data

**Fig. 1:**
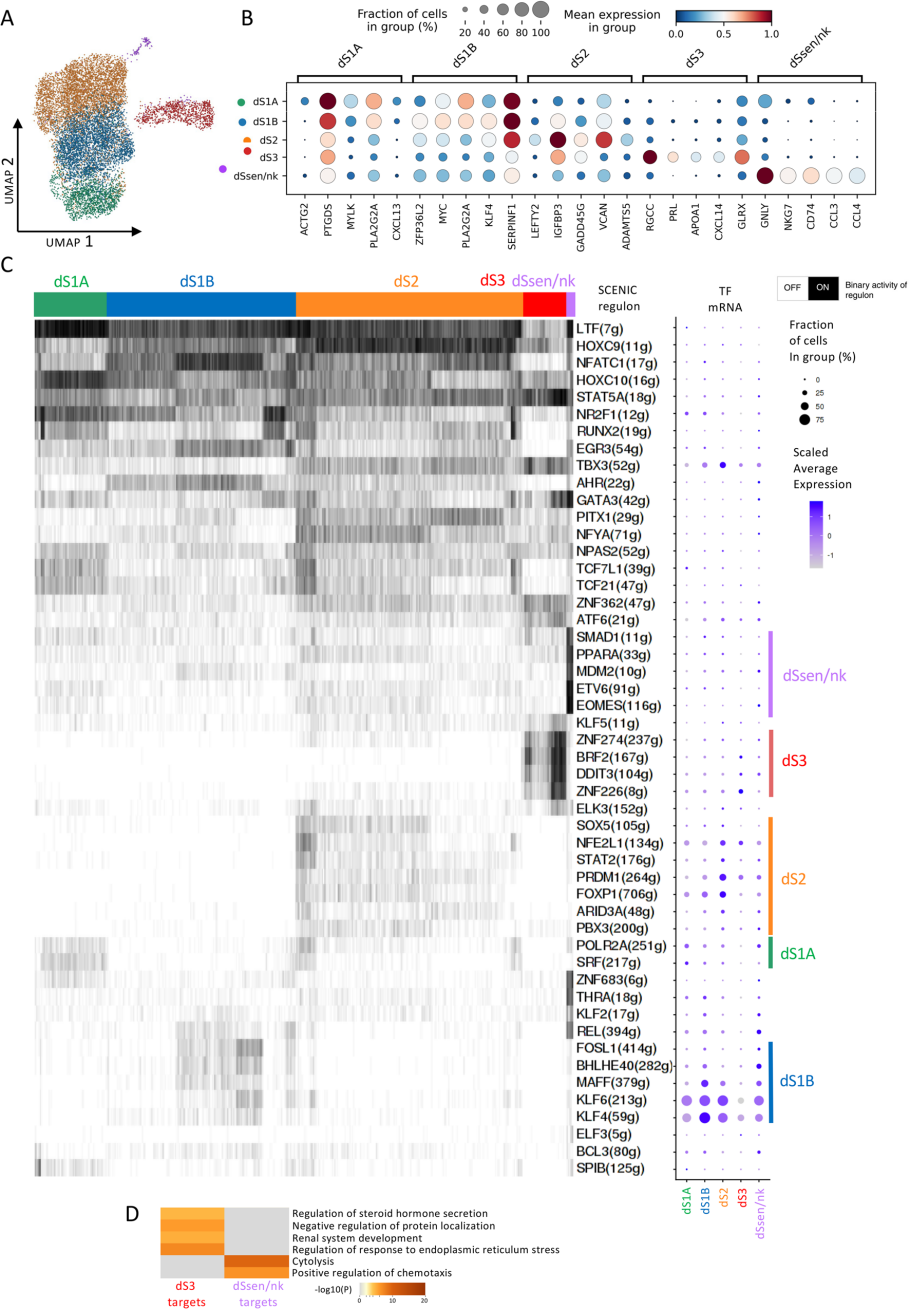
Gene regulatory network analysis of 1st trimester pregnancy decidual stromal (dS) cells. **A** UMAP visualization of the cluster analysis of the stromal single-cell transcriptomes, data from [1] **B** Top five maker genes of the stromal cluster with FDR 0.05 and FC ranking. dS1A and dS1B represent the undifferentiated fibroblastic state, dS2 the decidualizing state, dS3 the decidualized state and dSsen/nk the senescent/natural killer cell interacting cells. **C** A heatmap for single-cell gene regulatory network analysis (SCENIC) result for the top 10 cluster specific regulons (transcription factor + target genes). The column indicates subpopulation specific cell clusters (mRNA expression based) based and the rows indicate the regulon TF with number of target genes (g). See Supplementary Table S2 for regulon specificity scores. The dot plot displays the cell cluster specific mRNA expression of the TFs. On the right the selected cluster specific regulon TFs are marked with a bar. **D** Hierarchical clustering of selected enriched gene ontology (GO) terms for combined predicted dS3 regulon (BRF2, DDIT3, ZNF274 and ZNF226) targets versus predicted dSsen/nk regulon (SMAD1, PPARA, MDM2, ETV6 and EOMES) targets. See Supplementary Table S3 for detailed regulon target gene lists

We first re-analyzed the stromal subset (12,584 cells) from the 1st trimester selective termination scRNA-seq data from [1]. We identified five clusters instead of the three previously described clusters [1] (Fig. 1A). Our clustering analysis separated the original dS1 cluster into two distinct clusters, namely dS1A and dS1B, where dS1A consisted of the most fibroblastic type of stromal cells expressing ID2 and IGF1 (Supplementary Table S1). The decidualizing dS2 cluster expressed many classical pre-decidual markers such as LEFTY2 and FOXO1 (Fig. 1B, Table S1). Furthermore, the expression of prolactin (PRL) and IL1B confirmed the identity of the decidualized dS3 (DSC) cluster (Fig. 1B). However, recent studies in vitro have shown branching differentiation trajectories of decidual cells, resulting in a decidualized population and a senescent decidual population [5, 18], but this senescent population was not detected in the previous 1st trimester pregnancy data analysis. Here we identified a cluster with upregulation of senescence markers such as IL32 and CXCL8 (Fig. 1B, Supplementary Table S1), and apoptosis markers such as G0S2 (Supplementary Fig. 1), which were previously detected in assembloids in vitro [18]. This cluster also expressed several NK cell markers, including GNLY and NKG7. This suggests that these cells either express NK markers or alternatively these trancripts could originate from NK cells. Therefore, we annotated this cluster as senescence/NK associated decidual cells (dSsen/nk).

### Cluster Specific Transcriptomic Regulators of Decidual Stromal Cells

To gain insight into the major transcriptional regulators relevant to the 1st trimester stromal subpopulations, we performed a gene regulatory network analysis using SCENIC. We then displayed the top 10 stromal subpopulation-specific regulons, consisting of a TF and its predicted target genes (Fig. 1C, Supplementary Table S2). Using a heatmap depicting the binary regulon activity score for each cell, we identified whether the TF was active or not in a given cell. Our results revealed that a considerable proportion of the top 10 subpopulation-specific regulons were partially shared among the subpopulations. However, we also detected subpopulation-specific regulons (Fig. 1C). The subpopulation-specific activity scores of the top regulons were generally consistent with the scRNA-seq data, such that expression of the regulon TF itself peaked in the corresponding subpopulation. Furthermore, we observed that for many of the detected regulons, both the active regulon score and TF expression were only present in a fraction of the cells within the cluster. This might be partly due to incomplete detection of moderately or lowly expressed genes in scRNA-seq data.

For the undifferentiated fibroblastic subpopulations (dS1A, dS1B), we detected regulons such as POLR2A and SFR for dS1A, and FOSL1, BHLH40, MAFF, KLF6, and KLF4 for dS1B. In functional enrichment analysis, the predicted target genes in these regulons were associated with undifferentiated proliferative functions like "blood vessel morphogenesis" (p = 1.99 × 10^–9^) and "regulation of cell junction assembly" (p = 2.67 × 10^–9^) for dS1A, and "hematopoietic or lymphoid organ development" (p = 3.76 × 10^–22^) for dS1B (Supplementary Table S3).

### Regulon TFs and Networks for the Decidualizing Stromal Cells (dS2)

Next, we investigated the decidualizing stromal cell population dS2 in more detail. Our regulon analysis revealed a cluster of seven core regulons, which included PRDM1 (Blimp1), FOXP1, NFE2L1, SOX5, STAT2, ARID3A, and PBX3 (Fig. 1C and Supplementary Fig. 2). Of these regulons, especially the transcription factors PRDM1, FOXP and NFE2L1 had dS2 specific expression (Fig. 1C and Supplementary Fig. 2). Moreover, when the regulon activity on/off score and the expression of the TF itself was visualized with UMAPs, the regulon activity score of PRDM1, FOXP1 and NFE2L1 regulons matched more accurate than the TF expression with the cells in the dS2 subpopulation cluster (Fig. 1A and Supplementary Fig. 2). PRDM1, a known decidualization TF in mice [21], had several predicted target genes involved in decidualization, such as HAND2, LEFTY2, WNT5A, PRLR, and IGFBP2 (Supplementary Fig. 2, Supplementary Table S2). FOXP1 also has predicted targets LEFTY2 and PRLR, and additionally IL15, which has a crucial role in the stromal induced maturation of dNK cells [10]. NFE2L1 (NRF2), together with PRMD1, have as predicted targets decidualization genes such as follistatin (FST) [22], insulin receptors substrate 2 (IRS2) [23], and GADD45G (DDIT2). The functional enrichment analysis of the combined dS2 core regulons identified significant enrichment of GO terms such as “response to hormone” (p = 1.02 × 10^–22^) and “regulation of Wnt signaling pathway” (p = 3.01 × 10^–20^) (Supplementary Table S3).

**Fig. 2:**
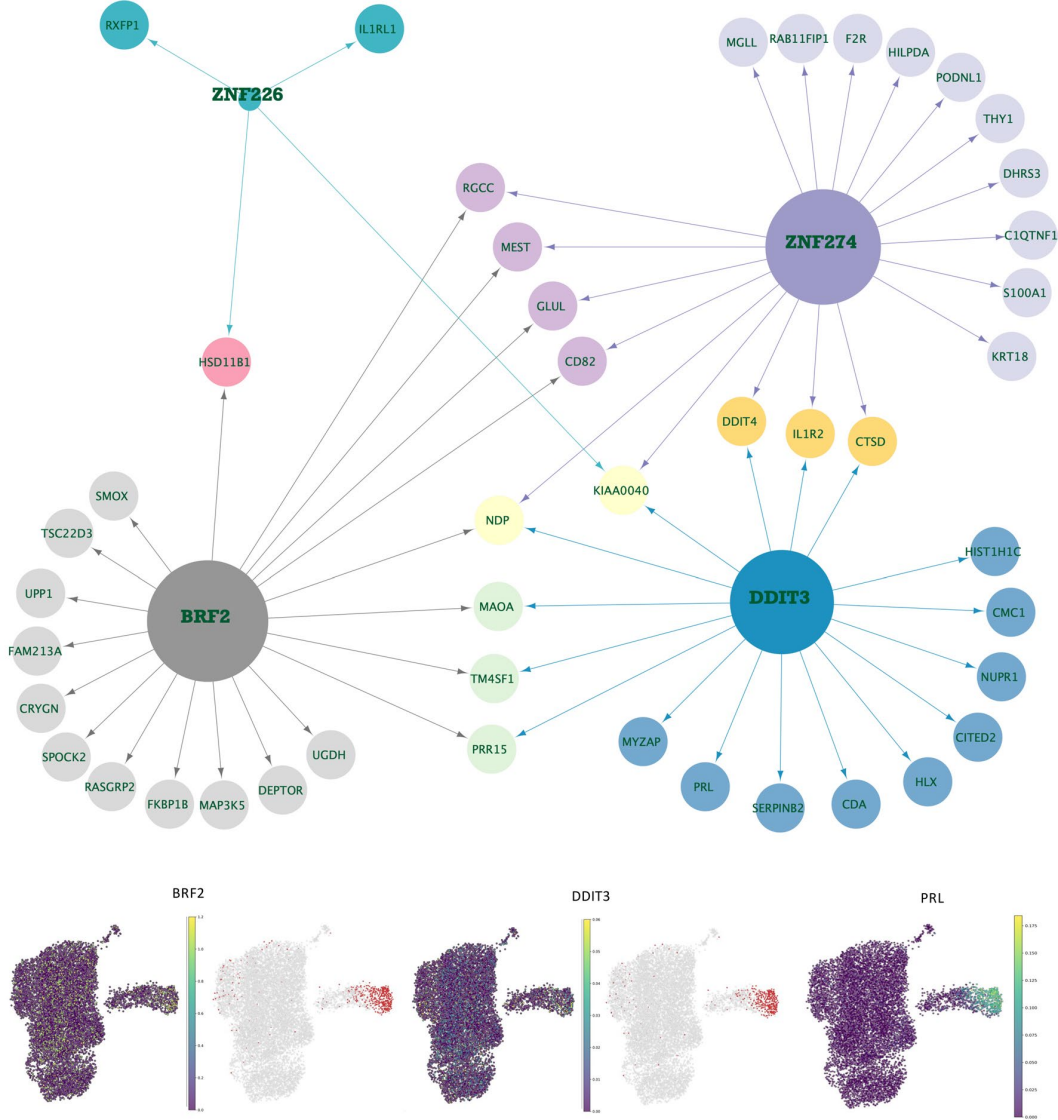
Gene regulatory networks of selected dS3 specific regulons. The size of the TF node is proportional to the number of target genes that the TF regulates, the target set is filtered to contain only cluster specific upregulated genes (FDR 0.05). The arrows indicate the target genes for the TF, and the different colors of target genes indicate target groups defined by the regulating TF or combinations of TFs. The UMAPs display the TF mRNA expression (left) and the binary regulon activity (on/off) (right) in each cell

### Regulon TFs and Networks for the Decidualized Stromal Cells (dS3)

For the decidualized stromal cell population dS3, we detected four core regulons, including DDIT3, BRF2, ZNF274, and ZNF226, of which the TF expression peaked in dS3 for DDIT3, BRF2, and ZNF226 (Fig. 1C). Notably, the cells where DDIT3 and BRF2 regulons were active showed the highest PRL expression (Fig. 2), confirming that their activity aligns accurately in vivo with cells that are decidualized, as indicated by the classical PRL decidualization marker. To our knowledge, these TFs have not previously been directly linked with decidualization, but their known functions align with the known decidual functions. DDIT3 is a known regulator of endoplasmic reticulum (ER) stress and unfolded protein stress responses [24] and a prolactin target gene in mouse decidua [25]. In our analysis, DDIT3 is predicted to target both decidual markers PRL, IL1R2, and DDIT4 (Fig. 2), as well as stress response proteins such as heat shock proteins HSPA5 (HSP70 family), and HSPB1 (HSP20 family) (Supplementary Table S3). BRF2 is a major redox-sensing transcription factor involved in oxidative stress protection [26]. Predicted BRF2 targets also included genes related to hormone-related stress responses such as HSD11B1, which is involved in the synthesis of the stress hormone cortisol. BRF2 and DDIT3 shared common predicted targets, such as MAOA, NDP, TM4SF1, and PRR15 (Fig. 2), of which monoamine oxidase MAOA is known to regulate the differentiation of endometrial epithelial cells [27], whereas NDP and TM4SF1 are part of wnt/beta-catenin signaling. BRF2 was also predicted to target secretory molecules, such as WNT4, typical to the secretory endometrium [28].

### Senescence/NK Associated Stromal Cells (dSsen/nk) are Predicted to be Targeted by NK Cells

For dSsen/nk, we identified five core regulons, including EOMES, ETV6, MDM2, PPARA, and SMAD1, of which TF expression peaked in dSsen/nk for EOMES and MDM2. EOMES is a known TF in NK cell differentiation [29] and regulates NK-mediated granulysis. The detection of several NK cell markers in this cluster, such as GLNY and NKG7 (Fig. 1B, Supplementary Table S3), supported the interpretation that mRNAs in the cells of this stromal cluster could partly originate from NK cells. Furthermore, NK cells are known to transmit protein and mRNA [3, 30]. Thus, because of the possibility of mRNA import, and the small number of cells in this cluster, we did not further focus on the GRNs of this cluster but conducted a functional enrichment analysis of the predicted core regulon targets to generally identify the functional differences between dSsen/nk and dS3. GO terms significantly enriched in dS3 but not dSsen/nk predicted targets included “regulation of response to endoplasmic reticulum stress” (p = 7.67 × 10^–8^) and “regulation of steroid hormone secretion” (p = 5.54 × 10^–7^) (Fig 1D, Supplementary Table S3), reflecting a transcriptional state of decidualized dS3, where these cells have acquired higher resistance for stress, in line with previous reports [4]. On the other hand, the dSsen/nk specific terms included “cytolysis” (p = 2.67 × 10^–10^) and positive regulation of chemotaxis (p 3.84 × 10^–7^) (Fig 1D, Supplementary Table S3). The detected “cytolysis” functional term for dSsen/nk is supported by the dSsen/nk specific upregulation G0S2 (Supplementary Fig. 1), that was recently reported as apoptosis inducer in decidualizing stromal cells [31], overall supporting the prediction that the dSsen/nk subpopulation is targeted for apoptosis and immunoclearance by the dNK cells [3].

### Reannotation of the dNK Clusters Using scRNA-seq Data

**Fig. 3:**
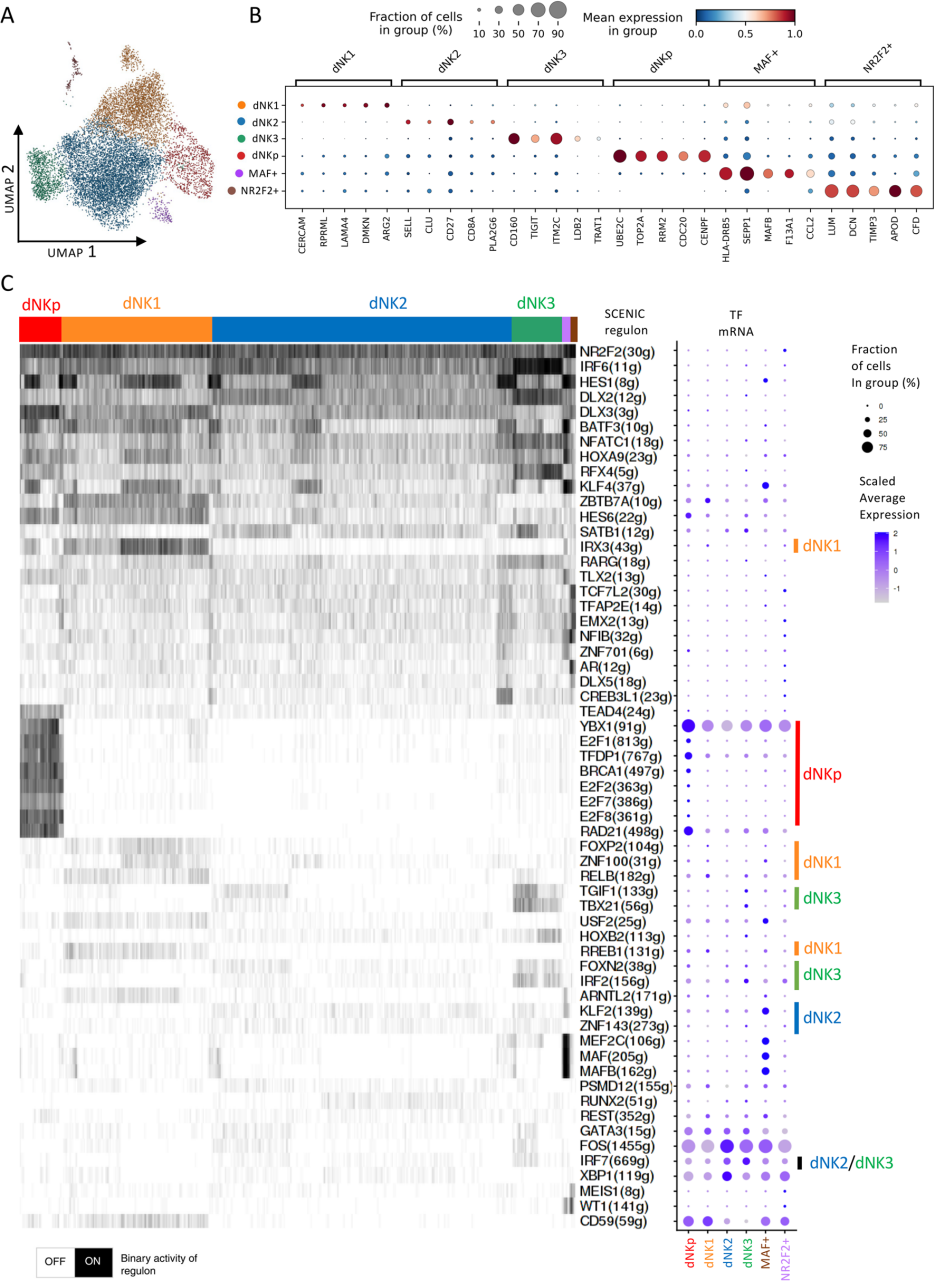
Gene regulatory network analysis of 1st trimester pregnancy decidual natural killer (dNK) cells. **A** UMAP visualization of the cluster analysis of the dNK single-cell transcriptomes [1] **B** Top five maker genes of the stromal cluster with FDR 0.05 and FC ranking. **C** A heatmap for single-cell gene regulatory network analysis (SCENIC) result for the top 10 cluster specific regulons (transcription factor + target genes). The column indicates subpopulation specific cell clusters (mRNA expression based) based and the rows indicate the regulon TF with number of target genes (g). See Supplementary Table S5 for regulon specificity scores. The dot plot displays the cell cluster specific mRNA expression of the TFs and on the right the selected cluster specific regulon TFs are marked with a bar. See Supplementary Table S6 for detailed regulons target gene lists

Next, we conducted a re-analysis of the dNK subset (11,884 cells) from the 1st trimester selective termination scRNA-seq data from [1]. We identified six clusters (Fig. 3A). Our cluster assignments for dNK1, dNK2, dNK3, and NK proliferative (NKp) were consistent with those from the original study [1]. Therefore, we adopted the same nomenclature. Our analysis also revealed two additional smaller adjancent clusters, which we annotated based on expression markers and regulon analysis (Fig. 3B) as dNK MAF + (155 cells) and dNK NRF2F2 + (134) clusters. The NRF2F2 + cluster had strong expression of typical stromal markers, but it clustered together with other dNK cell clsuters. For dNK MAF + cells the strong MAF transcriptional signature, a major macrophage marker, suggests that dNK MAF + cells either express macrophage markers or alternatively these transcripts could originate from macrophages. For both of these smaller adjacent clusters we did not proceed to full SCENIC analysis and focused on the major dNK clusters with > 1000 cells that are predicted to be functionally most relevant.

### Cluster Specific Transcriptomic Regulators of dNK Cells

**Fig. 4:**
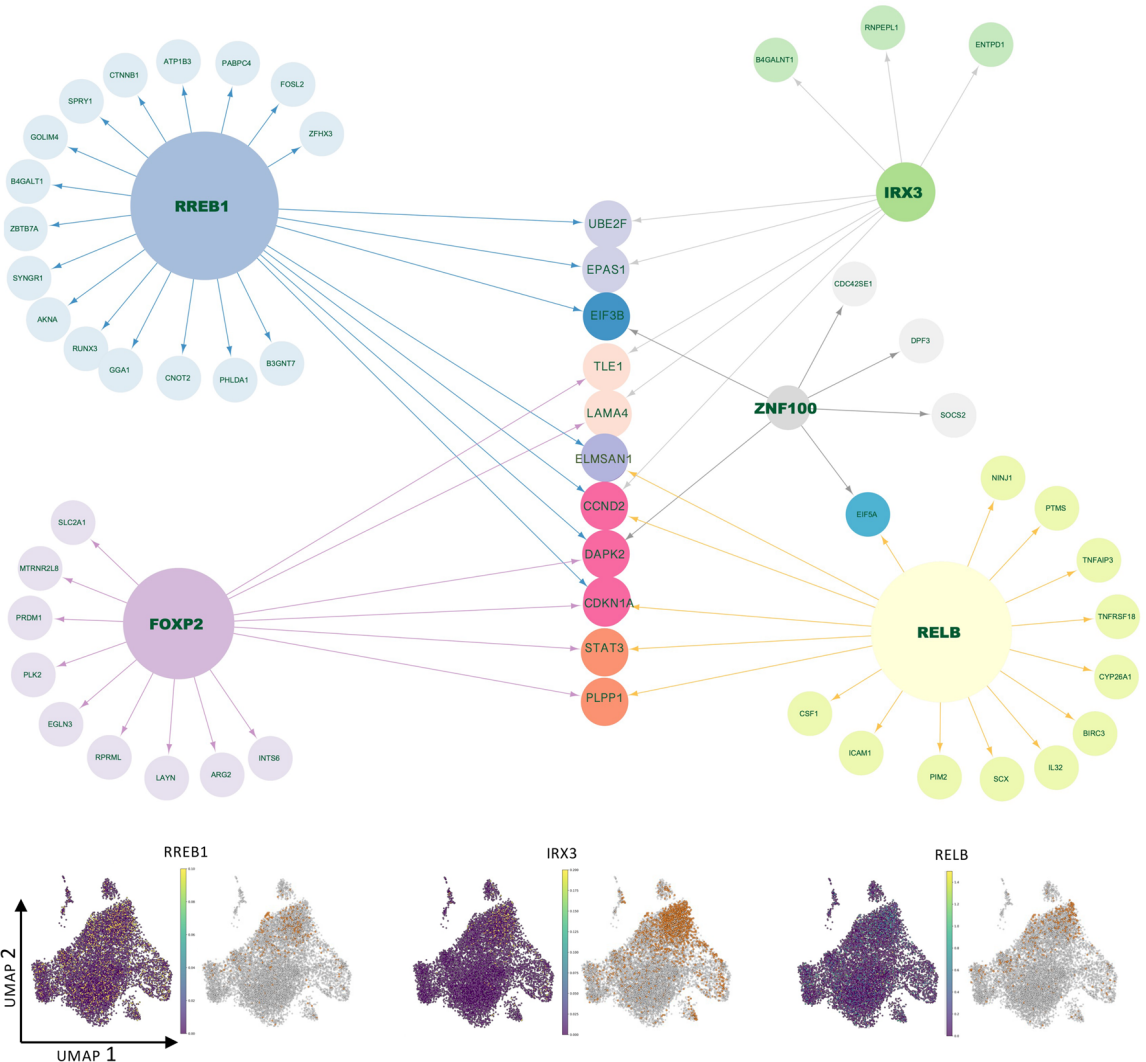
Gene regulatory networks and regulon activities of selected dNK1 specific regulons. The size of the TF node is proportional to the number of target genes that the TF regulates, and the target set is filtered to contain only dNK1 upregulated genes (FDR 0.05). The arrows indicate the target genes for the TF, and the different colors of target genes indicate target groups defined by the regulating TF or combinations of TFs. The UMAPs display the TF mRNA expression (left) and the binary regulon activity (on/off) (right) in each cell

**Fig. 5:**
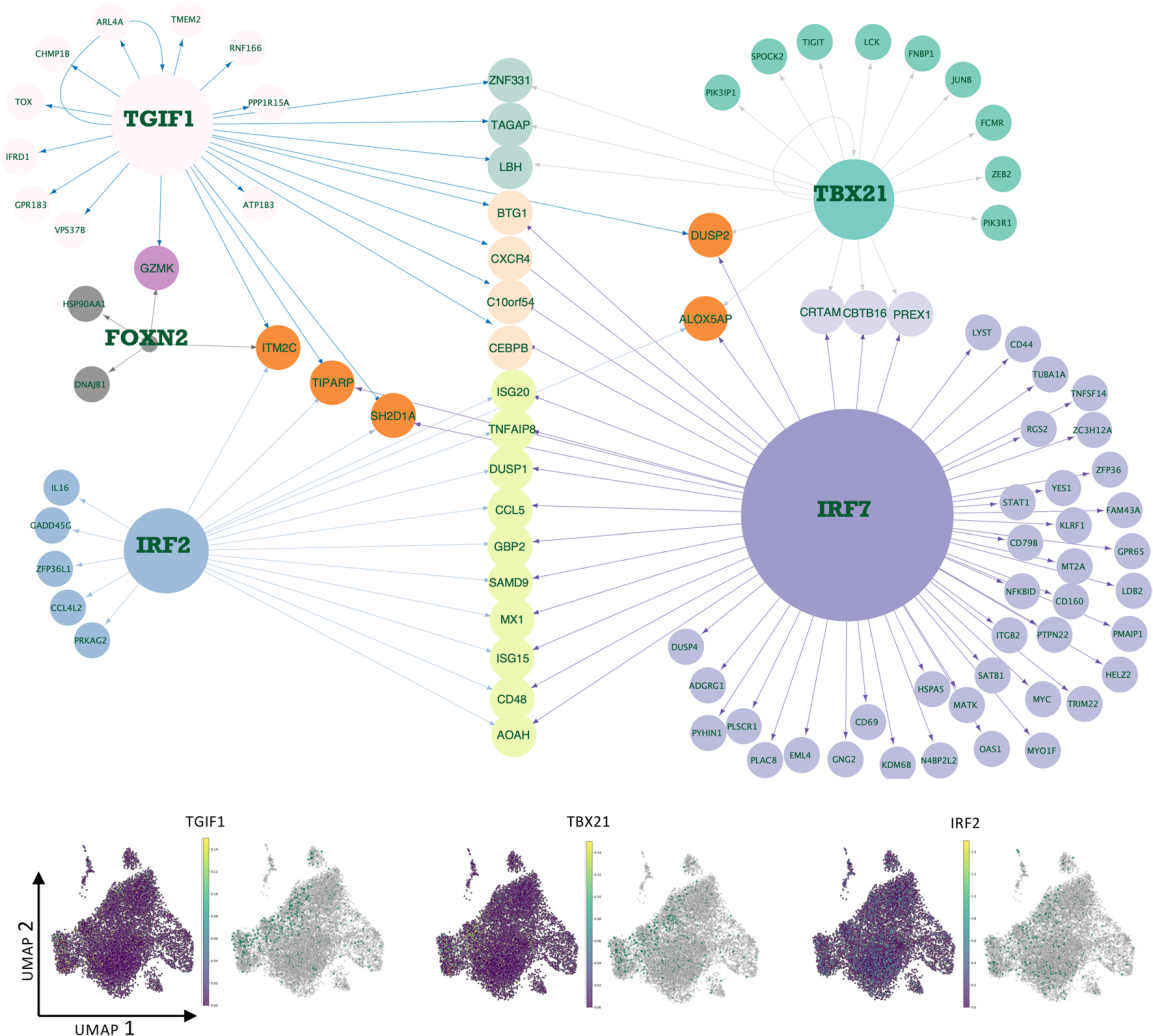
Gene regulatory networks and regulon activities of selected dNK3 specific or dNK2/dNK3 specific regulons. The size of the TF node is proportional to the number of target genes that the TF regulates, and the target set is filtered to contain only dNK3 upregulated genes (FDR 0.05). The arrows indicate the target genes for the TF, and the different colors of target genes indicate target groups defined by the regulating TF or combinations of TFs. The UMAPs display the TF mRNA expression (left) and the binary regulon activity (on/off) (right) in each cell

In order to understand the major transcriptional regulators specific to the 1st trimester dNK subpopulations, we ran SCENIC similarly as for the stromal cells and depicted the top 10 subpopulation-specific regulons using a heatmap (Fig. 3C). The dNKp population displayed a robust cell cycle regulator signature (E2F1, E2F2, E2F7 and E2F8). The regulon signatures for the other clusters were more subtle (Fig. 3C, Supplementary Table S5). Notably, in the dNK2 population, with the greatest number of cells, the vast majority of the regulons were shared with both dNK1 and dNK3. To emphasize the most relevant functional differences, we further investigated the divergent dNK1 and dNK3 subpopulations, with focus on the core subpopulation-specific dNK regulons where the mRNA expression of the regulon TF also peaked in the concordant subpopulation (Fig. 3C, 4 and 5).

In functional enrichment analysis, we observed that the predicted regulon targets of dNK1 included genes for development for the uterus and immunotolerance, whereas the predicted dNK3 regulon targets were enriched with inflammation related GO terms (Supplementary Table S6). Specifically, for dNK1, these included “in utero embryonic development” (p = 2.45 × 10^–9^) and “response to hormone” (p = 1.47 × 10^–7^), whereas the dNK3 top terms were “response to virus” (p = 9.33 × 10^–34^) and “defense response to symbiont” (p = 9.05 × 10^–26^) (Supplementary Table S6).

### Predicted dNK1 Regulon Targets Suggest that dNK1 Cells Promote Maternal Immunotolerance

The predicted dNK1 core regulons RELB, IRX3, FOXP2, and RREB1 and regulon targets that were up-regulated (FDR 0.05) in dNK1 cluster are displayed in Fig. 4. RELB is a negative regulator in NFKB pathway and is predicted to target other anti-inflammatory TFs such as STAT3. The RELB targets include TNFRSF18 receptor, CSF1, which promotes immunotolerance by interacting with extravillous trophoblasts [1, 32] and SPDL1, which reduces cytotoxicity of maternal immune cells [1, 33] (Supplementary Table S6).

IRX3 was detected as the top-ranked dNK1 specific regulator. It is predicted to target ENTPD1 (CD39), which has high dNK1 specific expression and was reported to convert extracellular ATP to adenosine [1, 34], thereby downregulating cytotoxic extracellular ATP buildup. In addition to direct immunomodulation, it has been reported that dNK1 cells are metabolically reprogrammed to induce glycolysis pathway [1]. IRX3 and RREB1 are predicted to target EPAS1 (HIF-2alpha), which is upregulated in dNK1, and HIFs are the known main glycolysis regulators. Moreover, predicted FOXP2 targets are enriched in GO terms such as “response to oxygen levels” (p = 1.38 × 10^–4^). Therefore, all three IRX3, RREB1 and FOXP2 may be involved in promoting metabolic reprogramming in dNK1.

Furthermore, RELB, RREB1 and FOXP2 all target CDKN1A, a senescence inducer, raising the possibility that dNK1 population or dNK cells, in general, not only survey stromal cells for senescence but also themselves are programmed for it.

### Predicted dNK3 Regulon Targets Suggest that dNK3 Cells Promote Inflammation

The predicted dNK3 core regulons TBX21 (T-bet), IRF2, TGIF1 and FOXN2, and predicted regulon targets that were upregulated (FDR 0.05) in dNK3 cluster are shown in Fig. 5. These regulons include TFs that are well-known regulators of NK development such as TBX21 (T-bet) and IRF2. TBX21 is predicted to target several genes related to “leukocyte migration” (p = 3.28 × 10^–7^) including CCR5, LCK, PIK3R1, SLAMF1, TBX21, CRTAM, PREX1 (Supplementary Table S6). TBX21 together with TGIF1 are predicted to target TAGAP, that is a known regulator of T cell differentiation and autoimmune diseases [35].

We observed that dNK2 and dNK3 shared multiple predicted regulons related to inflammatory signaling that promote NK cell cytotoxicity. These included transcription factors related to interferon signaling such as IRF1, IRF2, IRF5, IRF6, IRF6, IRF7 and IRF9 (Supplementary Table S5), with IRF7 being most notable for dNK2 and IRF2 for dNK3. The predicted targets of IRF2 and IRF7 included a wide range of genes involved in interferon response, such as IFNG and IFNA signaling mediators, cytokines such as CCL4, CCL5, CCL8, CCL19, XCL1, CXCL10, CXCR4, IL33, IL16 and CCL4L2, and core inflammatory regulators such as STAT1 and TNF (Fig. 5, Supplementary Table S6). IRF2 also regulated GADD45G, which has been reported to enhance IFNG production and anti-tumor immune effects [36].

In addition TGIF1 (TGFB Induced Factor Homeobox 1) was predicted to target genes involved in TGFB pathway (TGIF1, PPP1R15A, PMEPA1, GREM2) and genes in GO term “regulation of ERK1 and ERK2 cascade (p = 1.14 × 10^–5^) (Supplementary Table S6). Previously studies have shown that TGFB signaling is associated with the repression of NK-mediated cytotoxicity [37], suggesting the possibility of dNK3-specific TGFB-mediated immunotolerance mechanisms that are distinct from those indicated for dNK1.

### Pregnancy Disorder Downregulated Genes are Enriched Among Predicted dS2 / dS3 Regulon Target Genes and Disorder Upregulated Genes Among Predicted dNK2 / dNK3 Regulon Targets

**Fig. 6:**
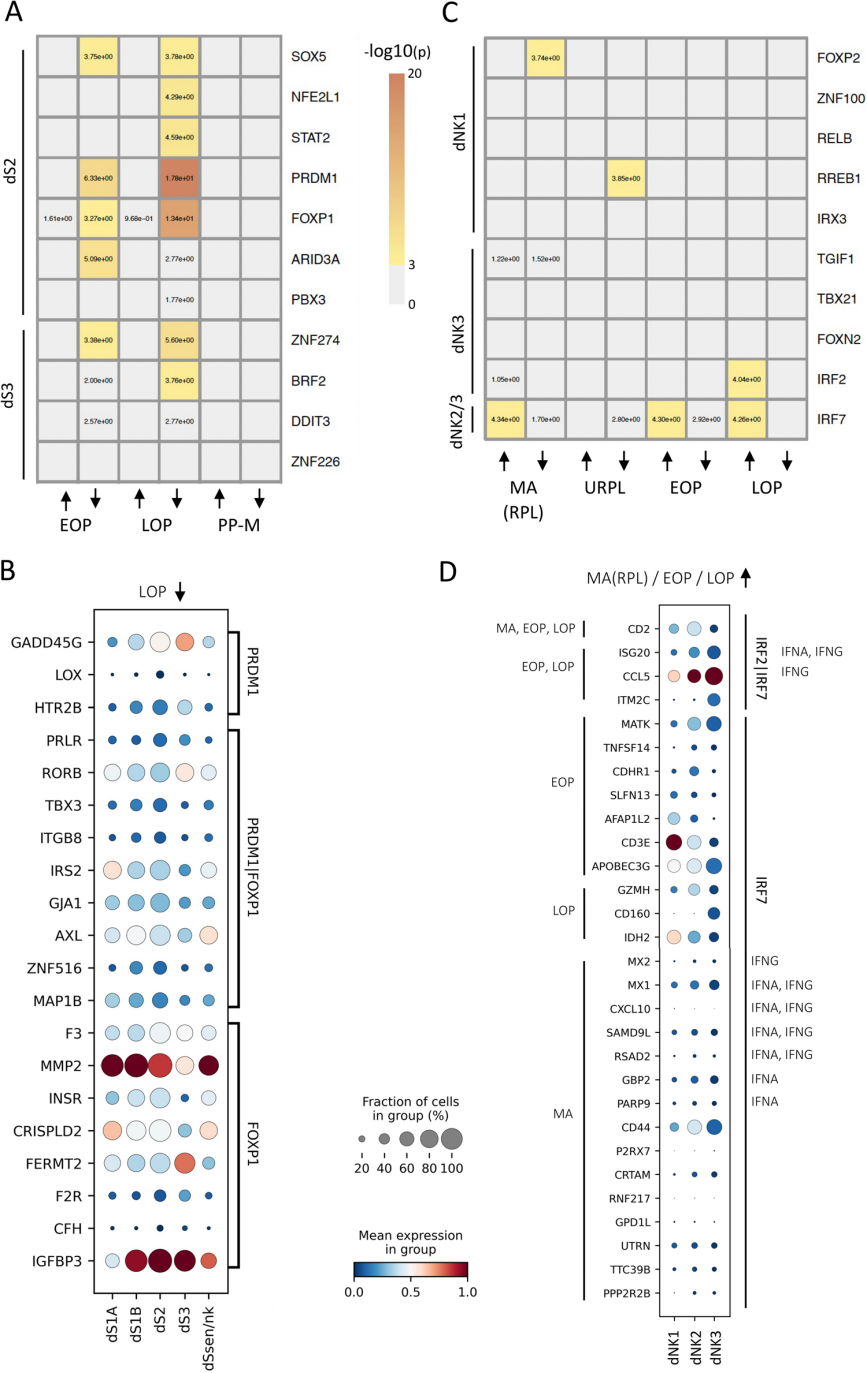
Overrepresentation analysis reveals pregnancy disorder associated cell subtype -specific regulons. **A** Overrepresentation of stromal dS2 and dS3 regulon target genes in pregnancy disorder datasets. The values in the cells are overrepresentation p values from Fisher’s exact test with all Refseq protein-coding genes (n = 20,203) as the background. The arrows on the x-axis represent up or down regulated disease genes in the corresponding bulk transcriptomic data. These included Early-Onset severe Preeclampsia (EOP) and Late-Onset severe Preeclampsia (LOP) from [20], and a study that contrasted women with previous severe preeclampsia to controls with normal pregnancy that were posteriorly collected during late secretory phase of Menstrual cycle (PP-M) [13]. **B** dS cell subpopulation specific mRNA expression of predicted PRDM1 and FOXP1 targets that were overrepresented in LOP downregulated genes. The specific PRDM1 and FOXP1 target groups are indicated on the right. **C** Overrepresentation of dNK1, dNK3, dNK2/3 regulon target genes in pregnancy disorder datasets. The values in the cells are p values from Fisher’s exact test with all Refseq protein-coding genes as the background. The arrows on the x-axis represent up or down regulated disease genes in the corresponding bulk transcriptomic data. These additionally included missed abortion (recurrent pregnancy loss) MA(RPL) data from [9], and unexplained recurred pregnancy loss enriched /underrepresented cluster specific genes (URPL) from [12]. **D** dNK cell subpopulation specific mRNA expression of IRF2 and IRF7 target genes that were overrepresented in MA(RPL), EOP or LOP upregulated genes. The condition in which the genes were upregulated is displayed on the left. On the right, IRF2 and IRF7 target groups are marked together with the specific classical IFNA or IFNG target genes. For the gene lists used as inputs and results of Fisher’s tests see Supplementary Table S7

We analyzed the translational relevance of the identified regulons by intersecting their predicted target genes with pregnancy disorder datasets. We utilized overrepresentation analysis to determine if the predicted regulon target genes were enriched among disorder upregulated or disorder downregulated genes (Fig. 6, Supplementary Table S7). The disorders analyzed included “severe preeclampsia, including early-onset (EOP, before week 34) and late-onset preeclampsia (LOP)”, “recurred pregnancy loss (RPL)”, or “missed abortion (MA)”. We found that for stromal cells, the predicted dS2 and dS3 specific regulon targets were enriched with preeclampsia downregulated genes, particularly regulon targets predicted for PRDM1 (LOP down p = 1.75 × 10^–18^, 21-fold) and for FOXP1 (LOP down, p = 3.8 × 10^–14^, 9.1-fold) (Fig. 6A, Supplementary Table S7). Figure 6B presents the mRNA expression of these predicted PRDM1 and FOXP1 targets in specific cell subpopulations. These include decidualization markers such as PRLR and IGFBP3, as well as genes that have previously been reported to have roles in trophoblast invasion, such as FERMT2 [38] and RORB, for which expression changes in preeclampsia have been observed in fetal placenta [39].

In dNKs, we observed that the predicted dNK2/3 regulon targets were enriched with disorder upregulated genes whereas the predicted dNK1 regulon targets were enriched with pregnancy disorder downregulated (/underrepresented) genes (Fig. 6C). For the pregnancy disorder upregulated regulons and target genes, we identified that the predicted dNK2/3-associated IRF2 and IRF7 targets were enriched with preeclampsia upregulated genes (IRF2 LOP p = 9.2 × 10^–5^, 19-fold; IRF7 EOP/LOP (p = 5.0 × 10^–5^/5.6 × 10^–5^), 7.1-fold / 8.6-fold) (Fig. 6C, Supplementary Table S7). Figure 6D illustrates the cell subpopulation-specific mRNA expression of these predicted targets, including CD2, ISG20, and CCL5, which are involved in proinflammatory responses. Notably, the cell surface receptor CD2, which mediates nanotube formation [40] and augments cytotoxicity [41], is a target of both IRF2 and IRF7 and was found to be upregulated in both preeclampsia and missed abortion. Furthermore, we found several predicted IRF7 targets enriched with missed abortion upregulated genes (p = 4.6 × 10^–5^, 3.7-fold) including classical interferon gamma and alpha response genes (CXCL10, GBP2, MX1, MX2, PARP9, RSAD2, SAMD9L) (Fig. 6D, Supplementary Table S7). These results support previous observations that upregulation dNK2/3 subpopulation specific transcriptomic states promote pregnancy disorders and emphasize the potential role of over-activated interferon pathway in the etiology of these disorders.

For the pregnancy disorder downregulated regulons and target genes, for dNK1, we found that that predicted FOXP2 targets were enriched with genes downregulated in missed abortion (RPL) [19], (p = 1.8 × 10^–4^, tenfold, LAYN, EGLN2, FOXP2, LAMA4 and SPARC) and predicted RREB1 targets were enriched with unexplained recurrent pregnancy loss underrepresented cluster [12] (p = 1.4 × 10^–4^, 36-fold, SYNGR1, CCND2 and UBE2F). Notably, LAYN, Layilin, is a receptor for hyaluronan (HA) [42], and decreased hyaluronan signaling may impair the NK mediated sensing of senescent decidual stromal cells and embryo quality, which has been previously observed [43]. Together, these result suggest that both the upregulation of inflammation associated dNK populations as well as downregulation of immunotolerance promoting dNK1 subpopulation contribute pregnancy associated disorders.

## Discussion

Recent single-cell transcriptomic studies have shown that subpopulations of decidual stromal cells and decidual natural killer cells play distinct functional roles during the first trimester of pregnancy [1]. However, the transcription factors that regulate these subpopulations have not been well characterized. In this study, we first re-analyzed the scRNA-seq clustering of dS and dNK cells and then used gene regulatory network analysis to investigate the core TFs for each dS and dNK subpopulation. Our analysis predicted both known TFs and novel TFs regulating the core subpopulations of stromal and dNK cells. The newly predicted TFs regulated several of the effector molecules that have been reported to be crucial for the subpopulations specific functions. Additionally, we predicted novel targets of potential functional significance. Finally, by integrative analysis of the predicted TF target gene sets with recent transcriptomic data from pregnancy disorders, we predicted subpopulation-specific regulator targets enriched in specific pregnancy disorder-regulated genes.

For stromal cells, we focused on the analysis of the dS2 and dS3 subpopulations, which are predicted to be the most crucial subpopulations for pregnancy [1]. For decidualizing dS2 cells, we detected top specific TFs to include PRDM1 (Blimp1), NFE2L1 (NRF2), and FOXP1. PRDM1 is a known decidualization factor in mice [21]. Our results predict that PRDM1 is a core regulator in human decidualization, regulating several genes that are part of progesterone and Wnt signaling. The predicted targets of PRDM1 also included HAND2 and FOXO1 that are known TFs involved in decidualization [6–8]. For dS3, the top regulators detected included DDIT3 and BRF2, which are predicted to regulate the expression of genes typical of secretory endometrium, but also notable genes involved in stress responses, including endoplasmic reticulum stress. DDIT3 itself is upregulated by prolactin [25] and both BRF2 and DDIT3 are known mediators of oxidative stress responses [26, 44].

Our findings align well with previous studies that have demonstrated stress-related regulation to be crucial for decidualization using in vitro experiments [4]. The described stress tolerance mechanisms include, for instance, oxidative stress resistance [45, 46], cessation of circadian rhythms [47], and uncoupling of SUMOylation [48]. Furthermore, analyses of the origin of decidual stromal cells indicated that several of the core decidualization regulators were originally mediating oxidative stress protection [49]. The findings of our study provide additional evidence to support the assertion that oxidative stress tolerance and other stress tolerance mechanisms are fundamental aspects of stromal decidualization.

For dNK subpopulations, the clustering analysis and our gene regulatory network analysis indicated that the major dNK subpopulations are closely related. However, we were able to predict subpopulation-specific transcriptional regulators for tolerance-type dNK1 and inflammatory-type dNK3, with functionally distinct target sets. The dNK1 specific TFs, such as IRX3, RELB, RREB1, and FOXP2, were predicted to regulate several genes that promote maternal immunotolerance. These predicted targets included previously observed immunomodulatory ligands, such as predicted RELB target CSF1 [1, 32], and enzymes involved in metabolic reprogramming, such as ENTPD1, a predicted target of IRX3, which converts extracellular ATP to less inflammatory adenosine.

In this study, IRX3 was identified as the top-ranked dNK1 specific regulator. In a previous study it was observed that IRX3 knockout in mice causes uterine defects, and this phenotype was associated with endothelial and stromal functions [50]. Our findings suggest that IRX3 may also be important in regulating the differentiation of dNK cell subpopulations. Additionally, IRX3 and RREB1 were predicted target hypoxia regulator HIF-2alpha (EPAS1), which may promote the dNK1 specific glycolytic program. NK cells have been observed to have augmented immunotolerance in hypoxic conditions (Solocinski et al. 2020), which is predicted to be enforced by glycolysis.

The regulators detected in dNK3 are associated with inflammatory signaling that promotes cytotoxicity, particularly via the interferon pathway, through transcription factors such as IRF2 and IRF7. Moreover, the dNK3-specific TBX21 (T-bet) is a classical NK regulator and is predicted to program NK cells for a more conventional cytotoxic and less tolerant transcriptomic state [9]. TBX21 is also involved in the terminal differentiation of NK cells (Zhang et al. 2021), and its specificity to dNK3 is consistent with the observation that during the third trimester of pregnancy, most dNK cells are of the dNK3 type, while NK1 and NK2 subpopulation proportions peak during the first trimester [51]. Additionally, it was previously observed that specifically dNK3 subpopulation exhibited relatively more transcriptomic changes between laboring and non-laboring states [51], suggesting that the dNK3 regulators and predicted targets identified in our study may also be involved in the onset of parturition.

Transcriptomic states associated with decidualization have been found to play significant roles in several pregnancy disorders, such as preeclampsia and recurrent pregnancy loss. Thus, we investigated the translational relevance of the identified cell subpopulation-specific core regulons in selected pregnancy disorder gene expression datasets. Overrepresentation analysis revealed that genes that are downregulated in preeclampsia were enriched with predicted decidualization-related stromal regulon targets. Specifically, predicted stromal cell dS2-specific PRDM1 and FOXP1 targets were enriched in preeclampsia downregulated stromal genes. These predicted targets included classical decidualization-inducing genes as well as genes previously reported to have preeclampsia-related transcriptional changes in trophoblasts, such as FERMT2 and RORB [38, 39]. These findings support previous observations that decidualization defects contribute to preeclampsia [13, 14, 52]. Furthermore, these results potentially suggest that stromal cells and trophoblasts share transcriptional changes associated with preeclampsia.

In the enrichment analysis of the predicted dNK cell subpopulation-specific regulon targets, the immunotolerance-supporting dNK1 regulon targets were enriched in genes downregulated in pregnancy disorders, while dNK3 and dNK2/3 regulon targets were upregulated in the same disorders. Notably, genes upregulated in preeclampsia and recurrent pregnancy loss were enriched with interferon pathway regulators IRF2 and IRF7, which promote NK cytotoxicity. For example, the cell surface receptor CD2 was upregulated in both disorders and is known to be involved in nanotube formation and increased cytotoxicity [40, 41].

When considering dS and dNK cells together in pregnancy maintenance (immunotolerance) and preparation for parturition, it is worth noting that the temporal differentiation of the cell subpopulations can be viewed as apparently opposite. For dS, in the differentiated dS3 population, the predicted TF targets that promote immunotolerance were downregulated in pregnancy disorder data. On the other hand previous studies point out that towards the end of pregnancy there are smaller proportion of differentiated dS3 cells detectable [1, 53], suggesting that the reduced dS3 proportion may be linked to parturition. In contrast, for dNK cells, the genes specific to the differentiated dNK3 cells were upregulated in pregnancy disorders, and it has been observed that towards the end of pregnancy, there are more dNK3 cells compared to dNK2 and dNK1 cells present [51], again, likely aligning with parturition onset. These opposing dynamics may be connected, for example, via stromal cytokines such as IL15 [10] or via dNK-mediated clearance of stromal cells [3, 8].

Overall, the strengths of our study include the systematic and detailed focus on the subpopulation specific TFs from in vivo scRNA- seq, and placing these carefully in the context of previous in vitro findings. These predictions can readily be utilized to generate testable hypothesis for experimentation. The limitation of this study is its predictive computational approach and limited sample sizes utilized in the analysis. For the predicted subpopulation specific TFs follow-up validations are required to further clarify their biological roles. Additionally, we did not follow up in detail the smaller adjacent subpopulations, but only focused on the major subpopulations consisting the majority of the studied cells.

As such, the TFs detected in this study constitute a physiologically relevant resource for future knockout studies in cell co-cultures [18] or mouse models [50]. For instance, for the immunotolerance promoting dNK1s, the top dNK1 specific transcription factor IRX3 knockout in mice [50] could provide an interesting avenue to investigate IRX3 also in the context of dNK function. This line of research could inform on the evolution of uterine dNK subpopulations in placental mammals. Previously the evolution of differentiated dS cell type has been studied [49], but for dNK cells studies with evolutionary aspects have not been conducted.

In conclusion, this study presented a comprehensive gene regulatory network analysis for decidual stromal and NK cells utilizing 1st trimester pregnancy single-cell transcriptome data. The study identified several well-known and novel transcriptional regulators for both stromal and dNK cells. Regarding stromal cells, the results emphasize the importance of stress resistance in decidualization and present novel factors, including BRF2 and DDIT3, for future mechanistic studies. For dNK cells, tolerance-type dNK1 and inflammatory-type dNK3 population-specific transcriptional regulators with functionally distinct target sets were predicted. The dNK1 regulators were predicted to target several anti-inflammatory pathways and metabolic effectors that may be responsible for tolerance-inducing metabolic programming, while dNK3 predicted regulators were predicted to target inflammation-inducing pathways, most notably the interferon pathway. Lastly, when the detected transcription factor target sets were studied using datasets of pregnancy disorders, we discovered a pattern of predicted tolerance-promoting regulator targets being downregulated in disorders and predicted inflammation-promoting targets being upregulated in disorders. These results provide a useful resource for future mechanistic studies of physiologically relevant in vivo regulators of decidualization.

## Supplementary Information

Below is the link to the electronic supplementary material.

Supplementary Table 1 (XLSX 45 KB)

Supplementary Table 2 (XLSX 47 KB)

Supplementary Table 3 (XLSX 239 KB)

Supplementary Table 4 (XLSX 528 KB)

Supplementary Table 5 (XLSX 58 KB)

Supplementary Table 6 (XLSX 170 KB)

Supplementary Table 7 (XLSX 115 KB)

Supplementary Fig 1.  (PDF 3.01 MB)

Supplementary Fig. 2.  (PDF 1.90 MB)

## Data Availability

The single-cell transcriptomic data underlying this article are available in ArrayExpress accession E-MTAB-6701. The pregnancy disorder data used is available in the supplementary tables of this article and in the cited original publications.
