# Supplementary material for: Gene Regulatory Network Analysis of Decidual Stromal Cells and Natural Killer Cells
Source: Reprod Sci. 2024 Aug 1;31(10):3159–74. doi: 10.1007/s43032-024-01653-1 (PMC11438719; doi:10.1007/s43032-024-01653-1)
Supplement: Supplementary file 8 — Supplementary Fig 1. (PDF 3.01 MB) [file 43032_2024_1653_MOESM8_ESM.pdf]

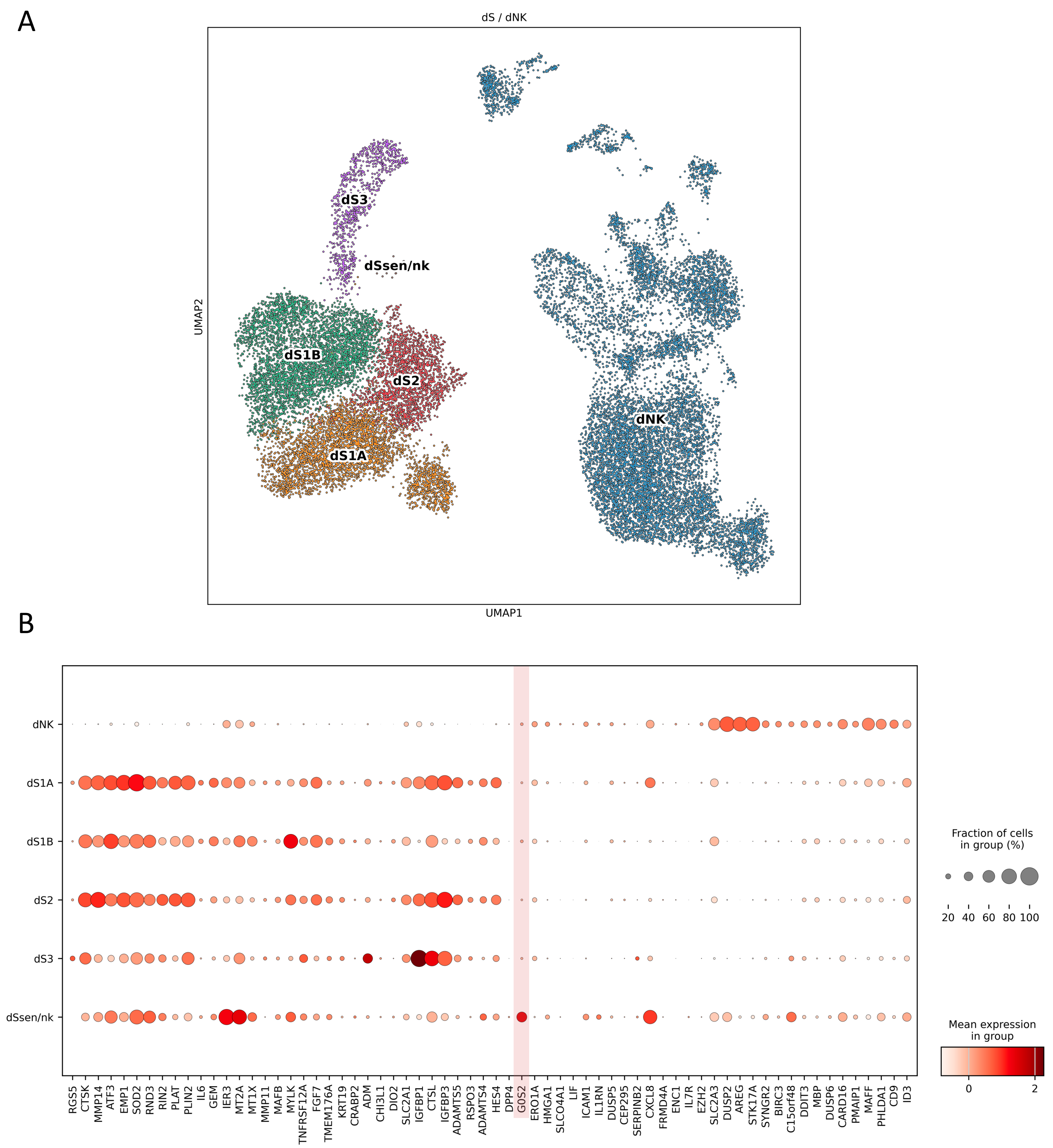

Supplementary Figure 1. Combined clustering of decidual stromal cells (dS) and decidual Natural Killer Cells. (A) UMAP1 differentiates clusters into dS clusters and dNK clusters. Subclusters of dS cells are annotated, and the adjacent stromal subcluster dSsen/nk a dS cluster. (B) Senescence markers from [18] for the clusters depicted (A). The expression for the known apoptosis inducer in endometrial stromal cells, G0S2 [31], is marked with light red background.
