# Supplementary material for: Gene Regulatory Network Analysis of Decidual Stromal Cells and Natural Killer Cells
Source: Reprod Sci. 2024 Aug 1;31(10):3159–74. doi: 10.1007/s43032-024-01653-1 (PMC11438719; doi:10.1007/s43032-024-01653-1)
Supplement: Supplementary file 9 — Supplementary Fig. 2. (PDF 1.90 MB) [file 43032_2024_1653_MOESM9_ESM.pdf]

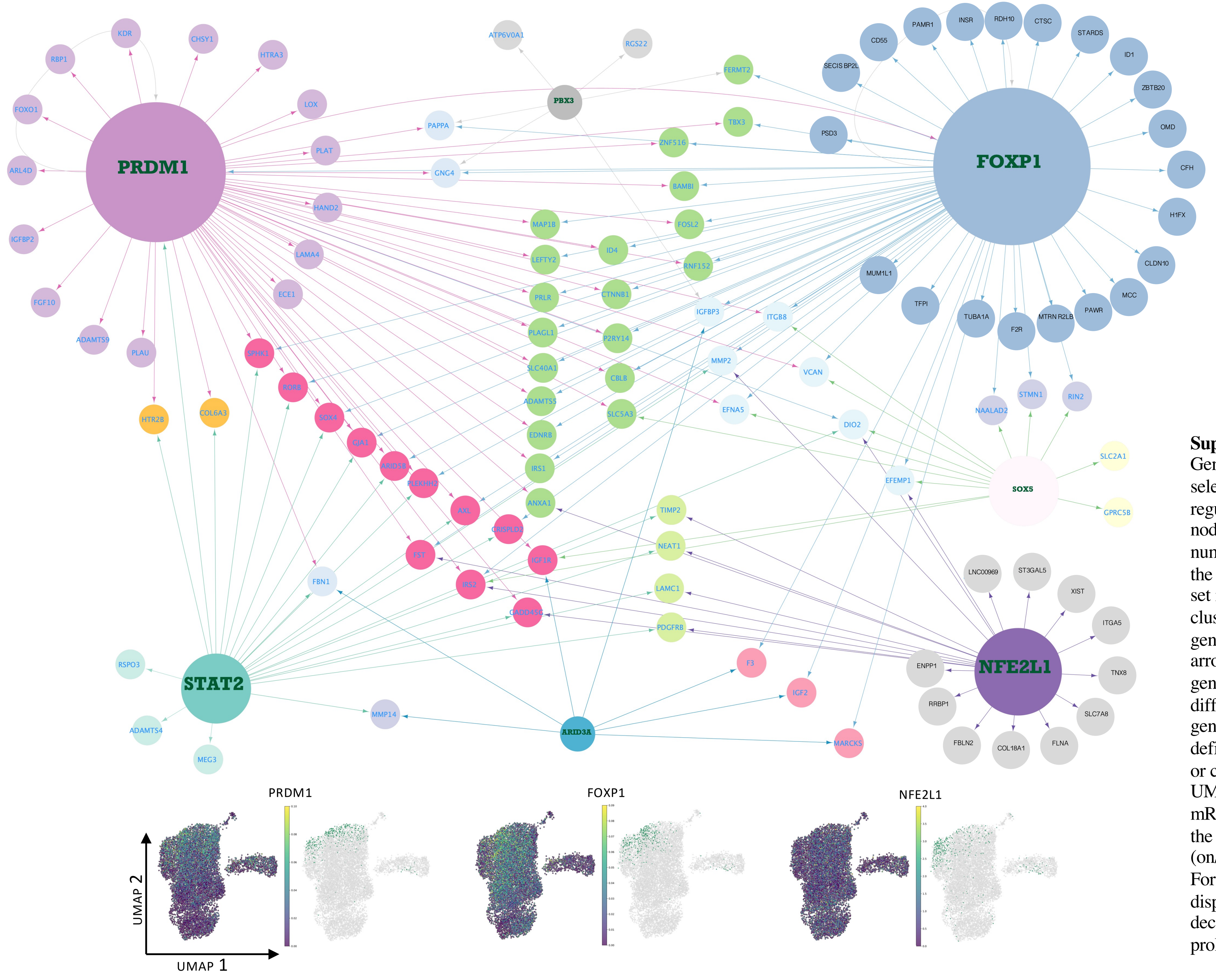

Supplementary Figure 2. Gene regulatory networks of selected dS2 specific regulons. The size of the TF node is proportional to the number of target genes that the TF regulates, the target set is filtered to contain only cluster specific upregulated genes (FDR 0.05). The arrows indicate the target genes for the TF, and the different colors of target genes indicate target groups defined by the regulating TF or combinations of TFs. The UMAPs display the TF mRNA expression (left) and the binary regulon activity (on/off) (right) in each cell. For (B) additional UMAP displays the expression decidualization marker prolactin (PRL).
